# Introduction to the Special Thematic Issue "Virtual Reality and Eye Tracking"

**DOI:** 10.16910/jemr.15.3.1

**Published:** 2024-06-02

**Authors:** Hasler Béatrice S., Rudolf Groner

**Affiliations:** University of Applied Sciences and Arts Northwestern Switzerland, School of Applied Psychology, Institute Humans in Complex Systems; University of Bern and SciAns Ltd. Iffwil

Technological advancements have made it possible to integrate eye tracking in virtual reality (VR) and augmented reality (AR), and many new VR/AR headsets already include eye tracking as a standard feature. While its application has been mostly limited to research in the past decade, we have now seen first installations of eye tracking into consumer level VR products in entertainment, training, and therapy.

The combination of eye tracking and VR creates new opportunities for end users, creators, and researchers alike: The high level of immersion – while shielded from visual distractions of the physical environment – has been shown to lead to natural behavior inside the virtual environment (16). This enables researchers to study how humans perceive and interact with three-dimensional environments in experimentally controlled, ecologically valid settings (3). Besides offering insights into human behavior, eye tracking in VR enables a variety of different practical use cases (1; 4).

Simultaneously, eye tracking in VR poses new challenges to gaze analyses and requires the establishment of new tools and best practices in gaze interaction and psychological research, from controlling influence factors, such as simulator sickness, to adaptations of algorithms in various situations.

This thematic special issue introduces and discusses novel applications, challenges and possibilities of eye tracking and gaze interaction in VR from an interdisciplinary perspective, including contributions from the fields of psychology, human-computer interaction, human factors, engineering, neuroscience, and education. It addresses a variety of issues and topics, such as practical guidelines for VR-based eye tracking technologies, exploring new research avenues, evaluation of gaze-based assessments and training interventions.

Due to the relative novelty of eye tracking in VR, there are no standard procedures and best practices established yet. Researchers and practitioners are examining use cases and pitfalls of the new technologies available on the market. In her article "Let’s get it started: Eye tracking in VR with the Pupil Labs Eye Tracking add-on for the HTC Vive" Josupeit (8) provides a practice example of an affordable VR eye tracking solution. In her step-by-step guide for novice users, she explains the hardware components and setup including the mounting of the eye tracker onto the lenses of the VR headset, and how to access the raw data, pre-process, visualize, and analyze the recorded eye tracking data using open-source software. In addition, she provides practical suggestions for experimental procedures. She stresses the advantages of opensource tools (in contrast to proprietary software) for the required transparency of the procedures and metrics in the reporting of VR-based eye tracking research in order to improve their reproducibility.

Eye tracking in VR has been explored for various types of training and appears to be particularly suitable for training visual motor skills. In their study "An investigation of feed-forward and feed-back eye movement training in immersive virtual reality" Harris et al. (6) evaluate how eye movement training (EMT) can be automated in VR to improve decision-making skills in military combat. The authors examined the relative benefits of observing an expert's gaze patterns (feed-forward EMT) compared to reviewing one's own eye movements to learn from one's own mistakes (feed-back EMT) in a virtual room clearance task. The experimenters randomly assigned military recruits to one of the EMT conditions and a control group that did not receive EMT in addition to their regular training. A post-test showed that both types of EMT resulted in more efficient gaze behaviors in the visual search of the virtual room, but did not improve speed and accuracy of decision-making. While these findings provide encouraging initial evidence for the benefits of VR-based EMT, the authors call for future research to examine whether performance improvements might require longer training durations or may be detected by using different performance measures.

In their article "Maintaining fixation by children in a virtual reality version of pupil perimetry" Portengen et al. (13) address the challenge of performing visual field examinations in young children. The study was designed to identify the best practices for optimal fixation and pupil response quality in a VR version of pupil perimetry. It tested three types of fixation tasks on healthy, three to eleven years old children: 1) fixating a dot, 2) watching an animated movie, and 3) counting the appearances of an animated character. While no significant differences were found between the fixation tasks regarding gaze precision and accuracy, the dot fixation and counting tasks resulted in the strongest pupil responses. The authors recommend the use of the counting task because children enjoyed it the most. The study shows that VR with integrated eye tracking is an ideal solution for visual field assessments in children as it provides accurate measurements and at the same time freedom of movement and engaging visual tasks.

Using eye tracking in VR for research and other use cases requires good tracking performance. Yet little is known about the data quality of eye trackers integrated into commercially available VR headsets. In their study "Eye Tracking in Virtual Reality: Vive Pro Eye Spatial Accuracy, Precision, and Calibration Reliability" Schuetz and Fiehler (15) systematically evaluate the eye tracking performance of the HTC Vive Pro Eye system. They tested participants with normal or corrected-to-normal vision (wearing contact lenses or glasses) across multiple sessions to evaluate calibration reliability and the influence of vision correction on spatial accuracy and precision of the recorded eye tracking data. The built-in calibration was reliable, irrespective of vision correction, but wearing glasses significantly reduced accuracy and precision. A significant difference in accuracy was observed between the tested (new and used) devices. Overall, the data quality appears to correspond with the device specifications. The authors report recommendations how to achieve this optimal performance level with the Vive Pro Eye system.

Eye tracking not only has many use cases in VR. Its potential has also been examined in combination with augmented reality (AR). Słowiński et al. (17)'s article "Assessment of cognitive biases in Augmented Reality: Beyond eye tracking" presents a novel AR method for the assessment of rational thinking and the heuristics (specifically, confirmation biases) that people employ when making decisions in complex environments. Participants completed a conventional questionnaire-based assessment of rational thinking and performed odd-one-out (OOO) tasks in AR with varying ambiguity that required them to select (i.e., touch) the object that is different (the "odd one"). Choices and response times as well as gaze, head and hand movements were recorded during the AR tasks. The authors found correlations between the questionnaire data and part of the behavioral measures. As one of the key findings, more rational thinkers had slower head and hand movements but faster gaze movements in the more ambiguous version of the OOO task. These findings illustrate how AR tools with integrated multimodal behavioral measures may be used to mitigate cognitive biases in various professional decision-making tasks, such as operators of defense and security systems, in aviation or surgery.

Eye tracking in VR further offers new possibilities to investigate cognitive mechanisms of visual perception. In their article "Flipping the world upside down: Using eye tracking in virtual reality to study visual search in inverted scenes", Beitner et al. (2) examined the disruptive effects of image inversion in naturalistic scenarios using an HTC Vive VR headset with a Tobii eye tracker. Participants completed an object search task using 3D virtual indoor scenes that were presented either upright or inverted (in randomized order within-subjects). Participants were slower in finding objects, moved their gaze more and made more fixations in inverted compared to upright scenes. Longer gaze durations helped them to find objects in inverted scenes faster. These findings illustrate how new insights can be generated by studying classical experimental paradigms using VR-based eye-tracking in naturalistic task settings under experimental control.

The article by Beitner et al. (2) demonstrates virtual reality with integrated eyetracking as a powerful new method for studying the human visual system. However, this approach requires the persistence of natural responses of the eyes to the (virtual) environment in VR displays. In the study "The pupil near response is short lasting and intact in virtual reality head mounted displays", Pielage et al. (12) experimentally examined the persistence of pupillary reflexes in VR; specifically, whether pupils show the expected constriction when virtual objects move closer to the user as they would in the real world. Greater pupil constriction was found to targets moving from far to near positions compared to targets moving from a far position to another far position. However, no significant pupil size changes were found when fixating targets that moved between positions at different distances. The authors conclude that pupil responses to targets at different distances are transient and do not result in sustained pupil size changes.

Observing moving objects creates a continuous following-movement of the gaze which is called smooth-pursuit. As only the observation of moving targets can evoke such smooth gaze movement, it can be used to infer which objects were observed and to base object selection on it. In their article "A systematic performance comparison of two Smooth Pursuit detection algorithms in virtual reality depending on target number, distance, and movement patterns", Freytag et al. (5) evaluate two algorithms that have been proven useful for the detection of smooth-pursuit-based object selection in 2D applications. These algorithms are adapted to include depth information and are applied to the detection of objects in 3D VR. The performance of both algorithms is compared under a variety of configurations of the target objects: movement patterns, object distances within the virtual space and the number of objects. In addition to suggestions for best practices, the authors discuss the concept of an adaptive threshold for the algorithms, which could support design and algorithm choices when designing 3D VR environments that include smooth-pursuit-based object selection.

In their article "Cognitive load estimation in VR flight simulator", Hebbar et al. (7) evaluate different methods to measure pilots' cognitive load when operating complex military aircraft interfaces. They used a VR-based simulation of the cockpit environment and artificial intelligence agents that acted as enemy aircrafts in different combat scenarios. As physiological measures of cognitive load, they recorded pupil diameter variations, gaze fixation rates and gaze direction using the HTC Vive Pro Eye headset with a built-in eye tracker and EEG signals. In addition, they collected subjective and performance-based measures of cognitive load. Both the eye tracking and EEG measures were good indicators of cognitive load variations and had strong associations with pilot's perceived task difficulty. Overall, the study shows that VRbased flight simulators are a promising method for developing and testing new aircraft technologies.

Overall, the articles of the present special thematic issue showcase a variety of use cases and applications of VR-based eye tracking in research and practice. They show how eye tracking in VR can be and has already been used to advance research in various fields, including research on the human visual system (2; 14) and cognitive processes ( 11; 17; 18), with important practical applications, for example in visual field examinations (13) and assessment of visual acuity ( 9; 10), military training (6) and for the development of new technological systems (7). Nevertheless, these articles also indicate there are still many open technical and methodological questions (5; 12; 15) that need to be addressed in future research to establish the required standards for scientific practice and an empirical basis for valid and reliable applications in various fields.

As editors, we hope this thematic special issue inspires further scientific inquiries in the rapidly converging fields of VR and eye movement research and sparks new ideas that consider the profound possibilities but also the challenges that lie at this exciting frontier.
